# Immunoglobulin G4‐Related Disease‐Like Gastric Lesion Presenting as Submucosal Tumor‐Like Lesions

**DOI:** 10.1002/deo2.70304

**Published:** 2026-02-21

**Authors:** Takayasu Kuroda, Kazuya Miyaguchi, Yoshikazu Tsuzuki, Hiroshi Yamaguchi, Hiroyuki Imaeda

**Affiliations:** ^1^ Department of Gastroenterology Saitama Medical University Saitama Japan; ^2^ Department of Pathology Saitama Medical University Saitama Japan

**Keywords:** endoscopic submucosal dissection, gastric lesion, IgG4/IgG ratio, IgG4‐related disease, submucosal tumor

## Abstract

Immunoglobulin G4 (IgG4)‐related disease (RD) is a systemic fibroinflammatory condition characterized by infiltration of IgG4‐positive plasma cells (PCs). Gastrointestinal involvement may present as submucosal tumor–like lesions and poses diagnostic challenges. We report a case of a 59‐year‐old man with gastric submucosal tumor–like lesions detected during routine screening. Endoscopic submucosal dissection (ESD) was used for the diagnosis and treatment. A histopathological examination revealed abundant IgG4‐positive PC infiltration in the submucosa; however, these findings did not fulfill the comprehensive diagnostic criteria for IgG4‐RD. IgG4‐RD–like gastric lesions can mimic submucosal tumors. ESD may provide diagnostic and therapeutic benefits when a conventional biopsy is insufficient. Cautious interpretation of IgG4‐positive PC infiltration in gastrointestinal specimens is required.

## 1 Introduction

Immunoglobulin G4 (IgG4)‐related disease (RD) is a novel systemic fibroinflammatory condition that was first reported in Japan by Hamano et al. in 2001 after they discovered elevated serum IgG4 levels in patients with autoimmune pancreatitis [1]. This novel disease has gained worldwide recognition as a distinct clinical entity.

Recently, the 2020 revised comprehensive diagnostic criteria for IgG4‐RD, which integrate clinical, serological, radiological, and pathological features to improve diagnostic accuracy across organ systems, including the gastrointestinal tract, have been proposed [2].

IgG4‐RD is characterized by tissue infiltration of IgG4‐positive plasma cells (PCs) that leads to fibrosis and progressive organ dysfunction. Pathological hallmarks of IgG4‐RD include storiform fibrosis, obliterative phlebitis, and marked infiltration of IgG4‐positive PCs in affected tissue [3]. Although the pancreas, bile ducts, lacrimal glands, and salivary glands are most commonly affected, IgG4‐RD can involve the gastrointestinal tract and manifest as esophagitis, ileitis, colitis, and proctitis. However, gastrointestinal involvement is rare [4].

IgG4‐RD with gastrointestinal involvement has been reported less frequently than IgG4‐RD with other organ involvement. We report a case of IgG4‐RD involving the gastrointestinal tract that manifested as submucosal tumor–like lesions and was diagnosed using endoscopic submucosal dissection (ESD).

## 2 Case Report

**FIGURE 1:**
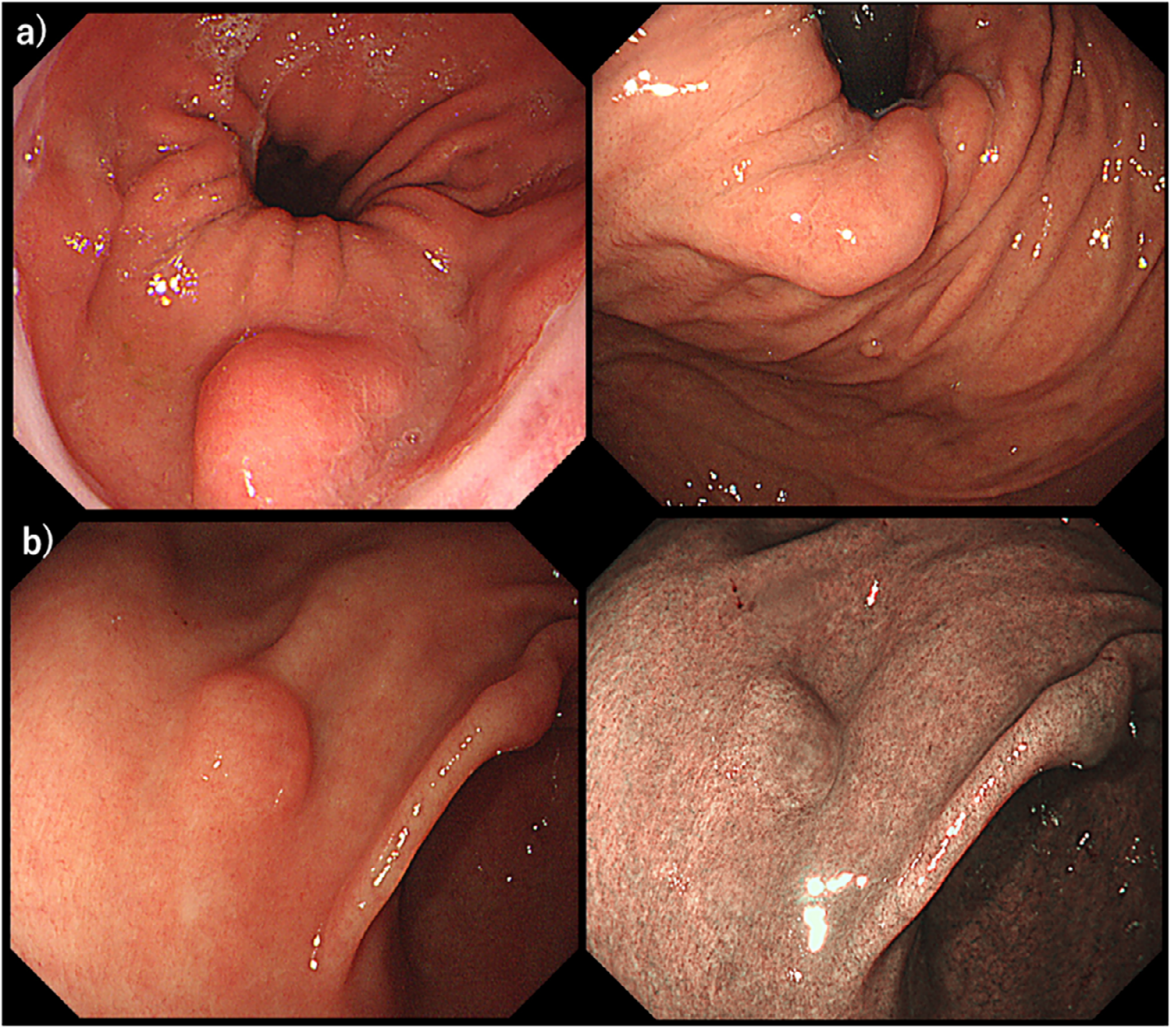
Upper gastrointestinal endoscopy findings. (a) The greater curvature of the gastric cardia observed with white light imaging (WLI) and (b) the anterior wall of the gastric antrum observed with WLI and narrow‐band imaging (NBI).

**FIGURE 2:**
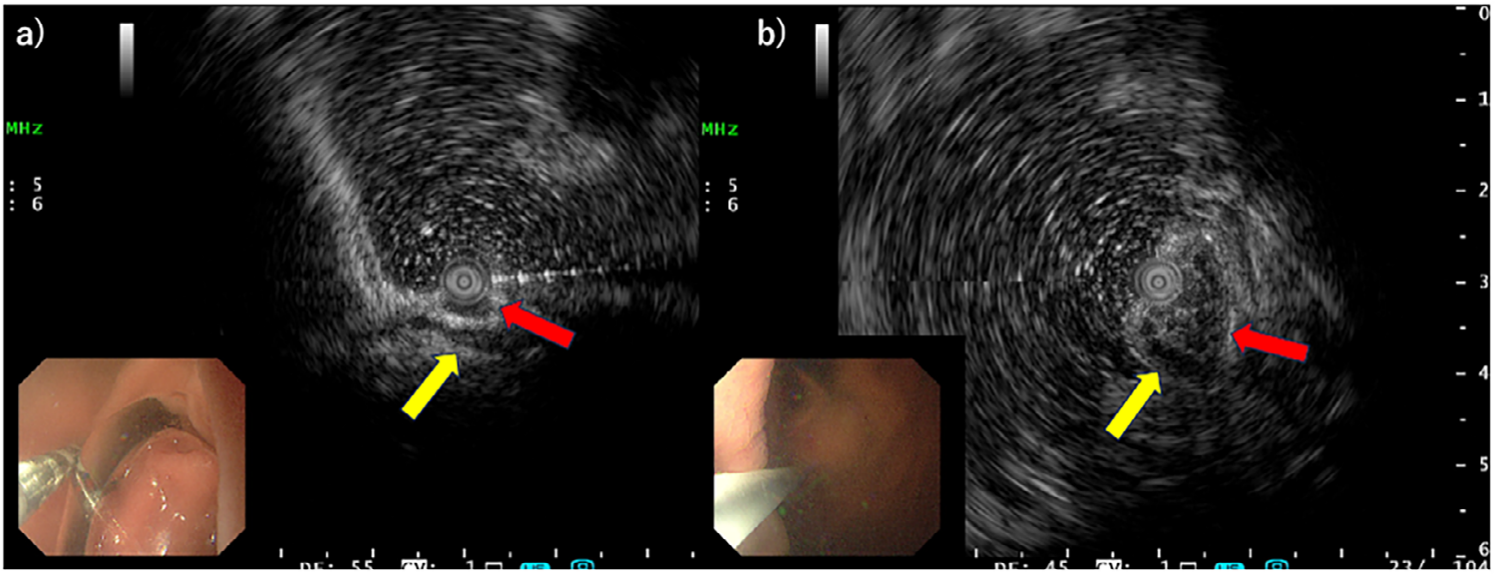
Endoscopic ultrasound images obtained using a 12‐MHz mini‐probe. The red arrow indicates the lesion. The yellow arrow indicates the fourth layer of the gastric wall. (a) Anterior wall of the antrum. (b) Cardia.

A 59‐year‐old man was referred to our department after a 1.6‐cm submucosal tumor in the gastric cardia was detected on upper gastrointestinal radiography. Upper gastrointestinal endoscopy showed no background mucosal atrophy but identified two lesions: a 10‐mm submucosal tumor–like lesion with a slightly depressed center and indistinct margins on the anterior wall of the gastric antrum and a 20‐mm submucosal tumor–like lesion on the greater curvature of the gastric cardia (Figure 1a,b). Endoscopic ultrasonography demonstrated a 6‐mm homogeneous hypoechoic lesion in the third layer of the anterior antrum and a 15‐mm heterogeneous hypoechoic lesion in the third layer of the cardia (Figure 2a).

**FIGURE 3:**
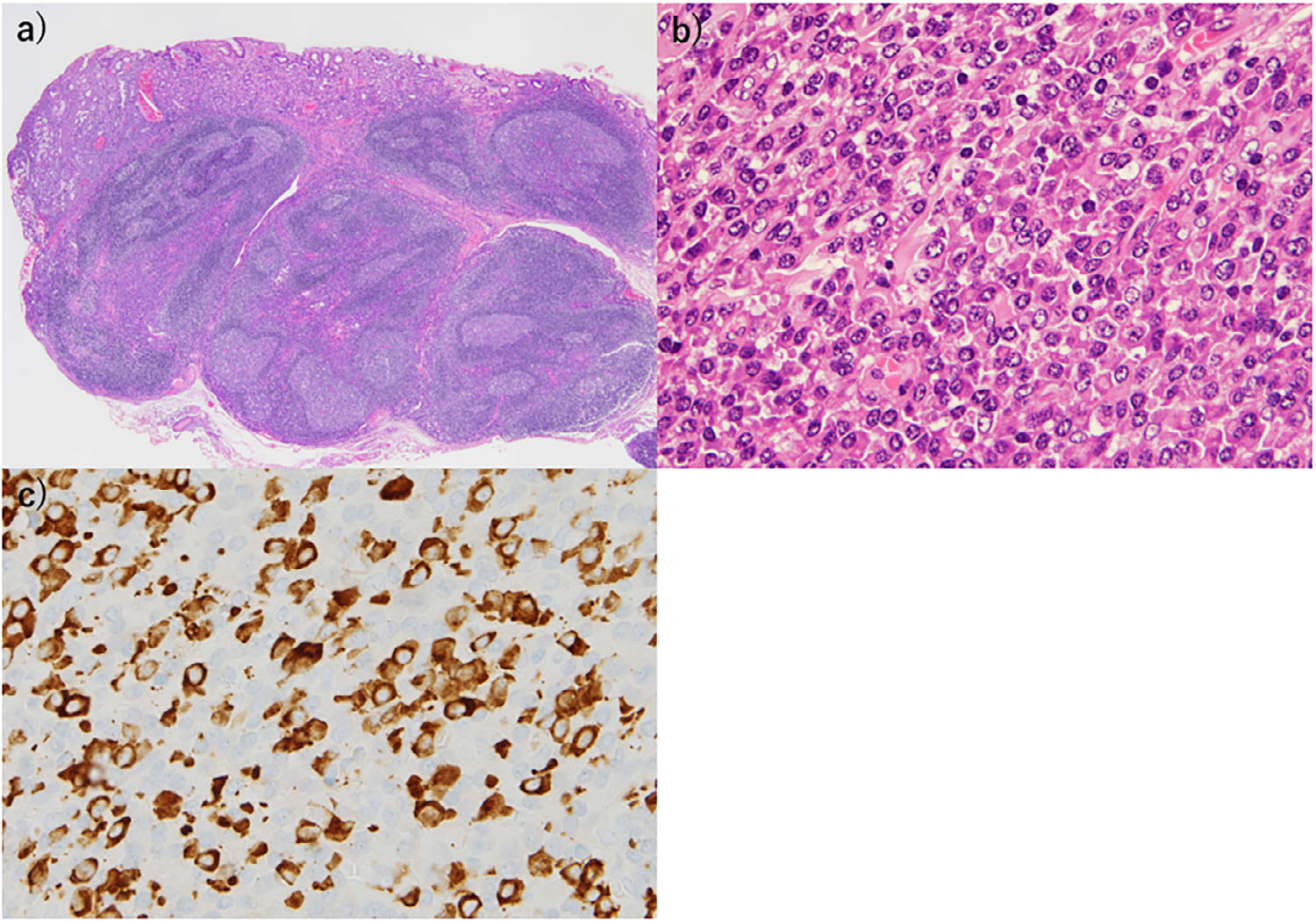
Histopathological findings. (a, b) The cardia and anterior antrum wall exhibiting lymphoid tissue with germinal center formation and atypical plasma cell infiltration, particularly in the interfollicular areas (×20 and ×40). (c) Immunohistochemical staining demonstrating numerous immunoglobulin G4 (IgG4)‐positive cells (×400).

Although the biopsy specimen was positive for synaptophysin and chromogranin A, a definitive diagnosis could not be established based on the biopsy findings alone. Therefore, the patient was admitted for ESD to further evaluate the lesions and establish a definitive diagnosis. A physical examination yielded unremarkable findings. Laboratory test results indicated elevated IgG levels (1882 mg/dL) with normal IgG4 levels; tumor markers were not significantly elevated. Contrast‐enhanced computed tomography demonstrated no pancreatobiliary involvement, lymphadenopathy, or other organ involvement suggestive of systemic IgG4‐RD. ESD of both lesions was performed. A histopathological examination of the fundic specimen revealed submucosal lymphoid tissue with germinal center formation and prominent interfollicular PC infiltration. An immunohistochemical analysis demonstrated CD20‐positive B lymphocytes within lymphoid follicles and polyclonal PCs expressing kappa and lambda light chains with markedly increased IgG4‐positive PCs. (Figure 3). Immunohistochemical staining revealed abundant IgG4‐positive PC infiltration with 80 IgG4‐positive cells per high‐power field (HPF) in some areas.

A histological examination revealed dense lymphoplasmacytic infiltration predominantly localized in the deep lamina propria that exhibited characteristic bottom‐heavy plasmacytosis. The superficial mucosa was relatively spared. Importantly, the muscularis mucosae was preserved, and the inflammatory infiltrate in the basal mucosa was clearly distinguishable from the underlying submucosal inflammatory pseudotumor; therefore, inflammatory cell spillover from the submucosa was excluded.

**FIGURE 4:**
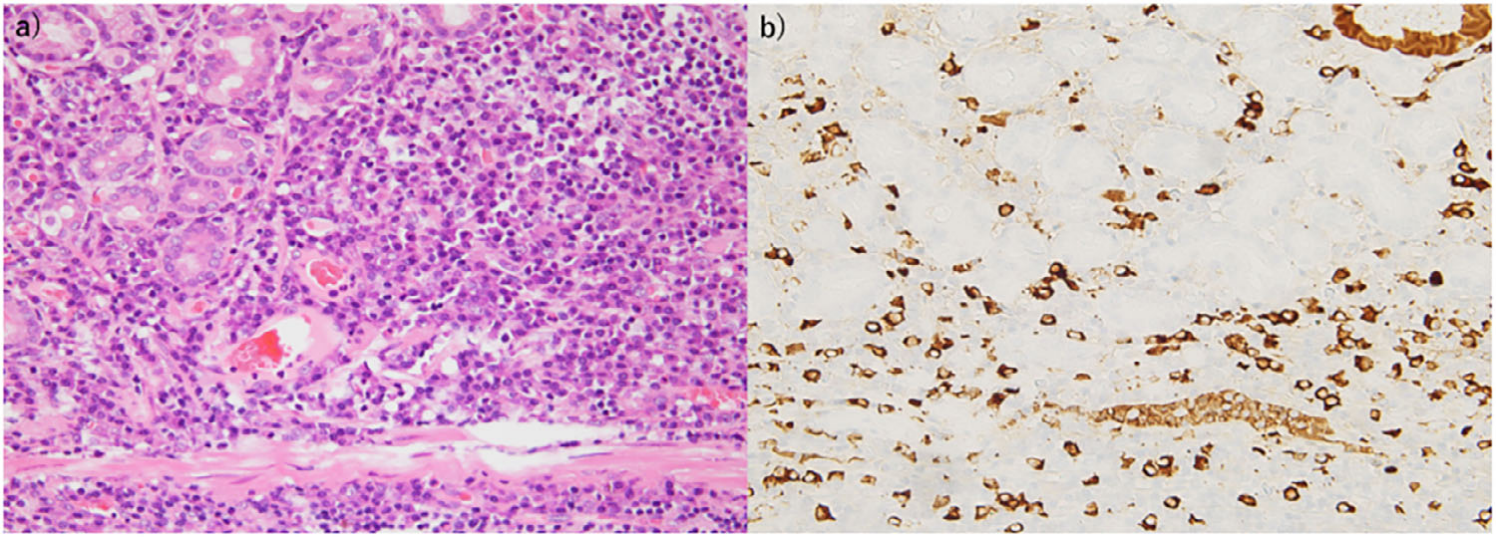
Histopathological features of an immunoglobulin G4 (IgG4)‐related gastric lesion demonstrating bottom‐heavy plasmacytosis. (a) Hematoxylin and eosin–stained section (original magnification ×200) showing dense lymphoplasmacytic infiltration predominantly in the deep lamina propria, consistent with bottom‐heavy plasmacytosis, with relative sparing of the superficial mucosa. (b) IgG4 immunohistochemistry (original magnification ×200) demonstrating numerous IgG4‐positive plasma cells with a corresponding bottom‐heavy distribution in the deep lamina propria.

An immunohistochemical analysis demonstrated numerous IgG4‐positive PCs with a similar bottom‐heavy distribution within the deep lamina propria, supporting the diagnosis of an IgG4‐RD–like gastric lesion (Figure 4). Inflammatory cell infiltration and IgG4‐positive cells were not observed in the mucosa surrounding the lesions. Based on these findings, the lesion was considered an IgG4‐RD–like gastric lesion rather than definitive IgG4‐RD. Although abundant IgG4‐positive PC infiltration and bottom‐heavy plasmacytosis were observed, the absence of storiform fibrosis, obliterative phlebitis, elevated serum IgG4 levels, and involvement of other organs indicated that the case did not meet the revised comprehensive diagnostic criteria for IgG4‐RD. After ESD, outpatient monitoring was conducted, and evidence of recurrence was not observed during 3 years of follow‐up.

## 3 Discussion

This case of IgG4‐related gastric lesions presented as submucosal tumor–like lesions and was successfully managed without corticosteroid therapy. Although most reported cases have been diagnosed surgically, this case demonstrates the use of ESD as a diagnostic and therapeutic approach, thereby avoiding invasive surgery. Notably, the presence of two submucosal tumor–like lesions in different gastric locations (cardia and antrum) is uncommon. This case further suggests that asymptomatic patients with isolated gastrointestinal involvement may be managed conservatively.

Two major patterns of IgG4‐related gastrointestinal lesions have been described: lesions with marked wall thickening caused by dense IgG4‐positive PC infiltration and fibrosis, and IgG4‐related pseudotumors in the stomach or colon (consistent with the present case) [5]. Chetty et al. reported IgG4‐related lesions presenting as nodular gastric elevations without autoimmune pancreatitis, as well as cases involving the cecum and sigmoid colon [6]. Additionally, Notohara et al. demonstrated that IgG4‐related gastrointestinal disease (RGD) may be suggested not only by dense IgG4‐positive lymphoplasmacytic infiltration but also by neural involvement of the muscularis propria and/or bottom‐heavy plasmacytosis in the gastric mucosa [7]. Bottom‐heavy plasmacytosis may support the diagnosis of IgG4‐RGD in an appropriate clinical context. In their series, IgG4‐positive PC counts ranged from 87 to 345 per HPF, whereas the IgG4/IgG ratio in our patient was only 7.0%. Nevertheless, this case was considered IgG4‐RD–like because of abundant IgG4‐positive PCs (>50/HPF in some areas), bottom‐heavy plasmacytosis, and clinical stability without treatment.

Standardized diagnostic criteria for IgG4‐RGD, particularly for isolated gastric involvement, have not been established. Despite suggestive histopathological features, this case did not meet all diagnostic criteria, reflecting the heterogeneous nature of IgG4‐RGD. A recent nationwide multicenter study further demonstrated the broad clinical spectrum and organ distribution of IgG4‐related gastrointestinal disease [8].

The diagnosis of IgG4‐RD is based on characteristic organ swelling or mass lesions, elevated serum IgG4 levels (≥135 mg/dL), and marked lymphoplasmacytic infiltration with fibrosis and IgG4‐positive PCs [9]. This case emphasizes that increased IgG4‐positive PCs alone are insufficient for diagnosis and that strict adherence to diagnostic criteria is essential to avoid overdiagnosis.

Steroid therapy is an effective first‐line treatment for IgG4‐related lesions [10]; however, careful diagnosis is required to avoid unnecessary surgery. IgG4‐related gastric lesions are rare, and most reported cases have been treated surgically, with no previous reports of ESD management.

IgG4‐RGD is rare and diagnostically challenging, particularly when presenting as submucosal tumor–like lesions. Integrated clinical, serological, and pathological assessments are necessary. In this case, en bloc resection was achieved using ESD without corticosteroid therapy because the patient was asymptomatic and had no other organ involvement. ESD represents a minimally invasive diagnostic and therapeutic option, although careful long‐term follow‐up is required because of the potential for recurrence or subsequent multiorgan disease.

## Author Contributions

**Manuscript drafting**: Takayasu Kuroda and Kazuya Miyaguchi. **Supervision**: Hiroshi Yamaguchi, Yoshikazu Tsuzuki, and Hiroyuki Imaeda. The final version of the manuscript was read and approved by all authors.

## Funding

The author has nothing to report.

## Ethics Statement

This case report was conducted in accordance with the ethical guidelines of our institution.

## Consent

Written informed consent was obtained from the patient for publication of this case report.

## Conflicts of Interest

The authors declare no conflicts of interest.
